# Enhancement of lignocellulosic degradation in high-moisture alfalfa via anaerobic bioprocess of engineered *Lactococcus lactis* with the function of secreting cellulase

**DOI:** 10.1186/s13068-019-1429-4

**Published:** 2019-04-17

**Authors:** Qinhua Liu, Junfeng Li, Jie Zhao, Jingxing Wu, Tao Shao

**Affiliations:** Institute of Ensiling and Processing of Grass, Nanjing Agricultural University, Weigang 1, Nanjing, 210095 China

**Keywords:** Cellulase, Ensiling, *Lactococcus lactis*, Lignocellulose

## Abstract

**Background:**

Butyric fermentation and a substantial loss of dry matter (DM) often occur in alfalfa silage during the rainy season, which is not conducive to subsequent biofuel production. Currently, there have been negative effects on the combination of cellulases and lactic acid bacteria (LAB) on processing high-moisture alfalfa silage; however, transgenically engineered LAB strains that secrete cellulase have been proposed as an alternative approach to avoid the above problem. The objective of the present study was to construct engineered *Lactococcus lactis* strains with high-efficiency secretory-expressing cellulase genes from *Trichoderma reesei* and to investigate the effects of the combination of transgenically engineered *L. lactis* strains HT1*/*pMG36e-*usp*45-*bgl*1, HT1*/*pMG36e-*usp*45-*cbh*2, and HT1*/*pMG36e-*usp*45-*egl*3 (HT2) on fermentation quality, structural carbohydrate degradability and nonstructural carbohydrate fermentation kinetics of high-moisture alfalfa silage treated without additive as a negative control (Control), or/and with cellulase (EN), wild-type *L. lactis* subsp. lactis MG1363 (HT1) and the combination of HT1 and EN (HT1 + EN) as positive additive controls.

**Results:**

Engineered *L. lactis* strains were successfully constructed and efficiently secreted endoglucanase (1118 mU/mL), cellobiohydrolase (222 mU/mL), and β-glucosidase (131 mU/mL) and had high filter paper activity (236 mU/mL). Ensiling experiments verified that HT2 obtained the highest fermentation quality score (83.6) and most efficiently processed high-moisture alfalfa silage, demonstrated by a low pH (4.49) and ammonia-N content (106 g/kg nitrogen) and a high lactic acid content (67.1 g/kg DM) and without butyric acid. Change curves of structural carbohydrates revealed that HT2 degraded more lignocelluloses, demonstrated by the lowest contents of neutral detergent fibre, acid detergent fibre, cellulose and hemicellulose after ensiling for 60 days. Kinetic analysis showed that the most residual water-soluble carbohydrates, glucose, fructose and xylose generated by lignocellulose degradation were produced by HT2, followed by HT1 + EN. The HT2-treated silages had the highest DM recovery, had the fewest *Clostridia* spores, emitted a fragrance and were not sticky.

**Conclusion:**

HT2 improved the conversion of lignocellulose to sugars and processed high-moisture alfalfa silage efficiently. This is a novel strategy that can be used to enhance lignocellulosic degradation in high-moisture alfalfa via a bioprocess with transgenically engineered *L. lactis* strains, which could enhance the development of alfalfa as a biomass feedstock and promote second-generation biofuel development in the rainy season.

**Electronic supplementary material:**

The online version of this article (10.1186/s13068-019-1429-4) contains supplementary material, which is available to authorized users.

## Background

The substitution of petroleum with renewable biomass feedstock for the production of chemicals and biofuel as a method to reduce greenhouse gas emissions and increase energy security has been given considerable attention [1]. Alfalfa is rich in protein, is widely used as animal feed and is also a good candidate feedstock for biofuel production [2].

Ensiling is a bioprocessing method for the anaerobic preservation and pretreatment of lignocellulosic biomass to produce feed and biofuel [3, 4]. However, alfalfa contains low contents of dry matter (DM) and water-soluble carbohydrates (WSC), especially in the rainy season, and has a high buffer capacity that often causes increased butyric acid and substantial DM loss [5]. The few nutrients in poor-quality alfalfa silage are not only useless for feeding animals but also decrease biofuel production due to insufficient soluble nonstructural carbohydrates, which deliver energy sources to yeast for producing bioethanol [6]. Moreover, high butyric acid concentration in poor-quality silage can restrain the growth of yeast [7]. Therefore, in the rainy season, maximally preserving high-moisture alfalfa via the ensiling bioprocess is an interesting and potential strategy for subsequent biofuel production.

Traditional methods for enhancing ensiling forage are wilting and adding acids, cellulase and lactic acid bacteria (LAB) alone or combined [5]. However, wilting alfalfa cannot be used in the rainy season, e.g., the plum rain season (from late spring to summer) in East China, and the enlarged cost and corrosivity are the disadvantages of using acids to ensile alfalfa [8]. Adding cellulase or LAB alone or the combination of cellulase and LAB have positive effects on improving lignocellulosic degradation and fermentation quality and reducing DM loss in many kinds of silages [9–11], but previous studies have described negative effects of the combined treatment of cellulase and LAB on alfalfa silage. Lynch et al. found that adding EN or LAB alone or combined did not affect DM losses or lactic and acetic acid contents of alfalfa silage compared with the control after ensiling for 70 days [12]. Kozelov et al. found that adding cellulase or LAB alone or combined had no effects on decreasing the contents of neutral detergent fibre (NDF) and non-fibre carbohydrates in high-moisture alfalfa silage; as a result, the silage had a high pH (> 5.0) after ensiling for 60 days [13]. The proposed explanation is that high-moisture alfalfa contains few WSC, some cellulases cannot degrade lignocellulose of alfalfa [12], or some lignocellulose degradation products of cellulase cannot be fermented by LAB [14, 15], e.g., high-molecular-weight oligosaccharides. Therefore, cellulase degrades lignocellulose into sufficient substrates which can be utilized by LAB to preserve high-moisture alfalfa as a biomass feedstock.

*Trichoderma reesei* is known as a potent cellulase producer because of its excellent genes that encode secreted cellulases, mainly endoglucanases, cellobiohydrolases and β-glucosidases [16], and the effective function of its cellulases to degrade lignocellulose into substrates utilizable by LAB during ensiling, as shown in our previous studies [17, 18]. To date, transgenic technology has allowed the expression of the endoglucanase gene of *T. reesei* in *Escherichia coli* to increase the level of expression of the endoglucanase gene using an inducible expression system, pET22b, and directed evolution [16, 19]. However, the total enzyme yields of recombinant *E. coli* were lower than the yields when using the signal peptide of Usp45 (*usp*45) derived from *L. lactis* and a pMG36e plasmid in *E. coli* (761 U/L) and *L. lactis* (1879 U/L), as described in our previous study [20]. One report concluded that *usp*45 could enhance secretion to increase heterologous protein production in *L. lactis* [21]. However, heterogeneous protein secretion in *L. lactis* using usp45 and pMG36e depends on many factors, e.g., the heterogenous protein size, conformation, and solubility [21–23]. The bigger size of cellobiohydrolase and β-glucosidase and their different conformations and solubilities from endoglucanase may hinder their secretion in *L. lactis* using usp45 and pMG36e. To date, little information is available on the successful expression of the cellobiohydrolase and β-glucosidase genes of *T. reesei* in LAB using the pMG36e plasmid and *usp*45. Therefore, clarifying the feasibility of successfully constructing transgenically engineered LAB that secrete endoglucanase, cellobiohydrolase and β-glucosidase using the pMG36e plasmid and *usp*45 benefits second-generation biofuel development, because it is increasingly dependent on the extracellular expression of cellulases [24]. Their application in ensiling may produce lignocellulose degradation products utilizable by *L. lactis* to promote lactic fermentation and provide an opportunity to better develop alfalfa as an alternative biomass feedstock for second-generation biofuel development in the rainy season.

The aim of this study was to construct transgenically engineered *L. lactis* strains that secrete endoglucanase, cellobiohydrolase, and β-glucosidase by a simple and highly efficient secretory expression method and investigate the effect of the combination of transgenically engineered *L. lactis* strains HT1*/*pMG36e-*usp*45-*bgl*1, HT1*/*pMG36e-*usp*45-*cbh*2, and HT1*/*pMG36e-*usp*45-*egl*3 (HT2) on processing high-moisture alfalfa as biomass feedstock via the evaluation of fermentation quality, structural carbohydrate degradability and nonstructural carbohydrate fermentation kinetics, using no additive as a negative control (Control), and cellulase (EN), wild-type *L. lactis* subsp. *lactis* MG1363 (HT1) and the combination of HT1 and EN (HT1 + EN) as positive additive controls.

## Methods

### Bacterial strains, plasmids and culture conditions

**Fig. 1:**
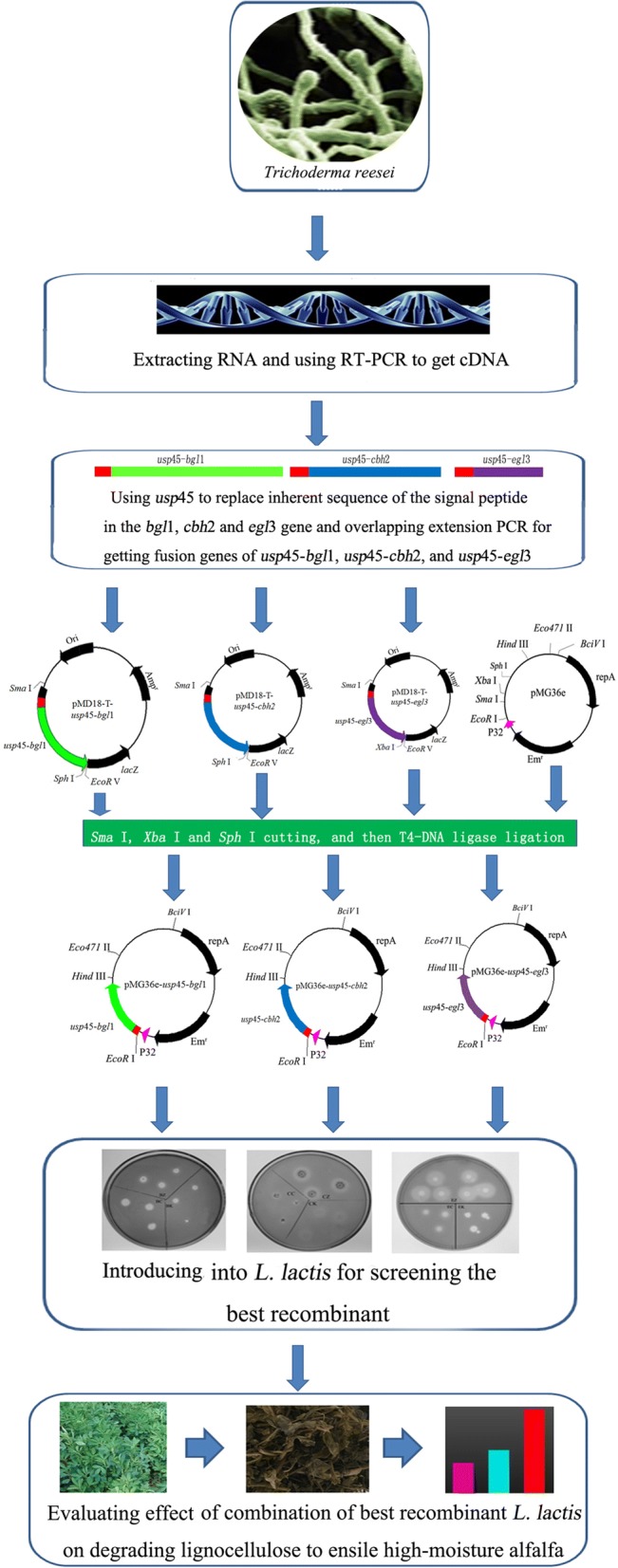
Experimental schematic

The bacterial strains and plasmids used in this study are listed in Table 1. *Escherichia coli* DH5α (DH5α) was cultured in Luria–Bertani medium (both agar and broth) at 37 °C, and HT1 was grown in M17 broth (Oxoid Ltd., Shanghai, China) supplemented with 5 g/L glucose (GM17) at 30 °C. A total of 300 μg/mL of erythromycin (Takara Biotechnology Co., Ltd., Dalian, China) was used to screen the positive DH5α recombinants containing the pMG36e backbone, and 5 μg/mL of erythromycin was used to screen the positive recombinant HT1 containing the pMG36e backbone. 40 μg/mL of ampicillin (TakaRa Biotechnology Co., Ltd., Dalian, China) and 4 μL of 100 mg/mL IPTG (isopropyl β-d-1-thiogalactopyranoside) (TakaRa Biotechnology Co., Ltd., Dalian, China) were plated on agar to screen for the positive DH5α recombinants containing the pMD18-T backbone*. T. reesei* 3.3711 (China General Microbiological Culture Collection Center) was cultured in potato dextrose broth or agar (Nissui-seiyaku Ltd., Tokyo, Japan) medium at 30 °C for 72 h. The experiment was carried out according to the schematic (Fig. 1).

**Table 1:** Bacterial strains and plasmids used in this study

Strain and plasmid	Relevant trait(s)	Source or reference
Strains		
*Escherichia coli* DH5α	supE44 Δlac U169 (Φ80 lacZ ΔM15) hsdR17 recA1, endA1 gyrA96 thi-l relA1	This laboratory
*Lactococcus lactis* subsp. *lactis* MG1363	A plasmid-free derivative of NCDO712; source of *usp*45; ADT indicator	This laboratory
*Trichoderma reesei* 3.3711	Type culture	China General Microbiological Culture Collection Center
*E. coli* DH5α*/*pMG36e	*E. coli* DH5α with pMG36e	This study
*E. coli* DH5α*/*pMG36e-*bgl*1	*E. coli* DH5α with pMG36e-*bgl*1	This study
*E. coli* DH5α*/*pMG36e-*cbh*2	*E. coli* DH5α with pMG36e-*cbh*2	This study
*E. coli* DH5α*/*pMG36e-*egl*3	*E. coli* DH5α with pMG36e-*egl*3	This study
*E. coli* DH5α*/*pMG36e-*usp*45-*bgl*1	*E. coli* DH5α with pMG36e-*usp*45-*bgl*1	This study
*E. coli* DH5α*/*pMG36e-*usp*45-*cbh*2	*E. coli* DH5α with pMG36e-*usp*45-*cbh*2	This study
*E. coli* DH5α*/*pMG36e-*usp*45-*egl*3	*E. coli* DH5α with pMG36e-*usp45*-*egl*3	This study
*E. coli* DH5α*/*pMD18-T-*usp*45-*bgl*1	*E. coli* DH5α with pMD18-T-*usp*45-*bgl*1	This study
*E. coli* DH5α*/*pMD18-T-*usp*45-*cbh*2	*E. coli* DH5α with pMD18-T-*usp*45-*cbh*2	This study
*E. coli* DH5α*/*pMD18-T-*usp*45-*egl*3	*E. coli* DH5α with pMD18-T-*usp*45-*egl*3	This study
*L. lactis* subsp. *lactis* MG1363/pMG36e	*L. lactis* subsp. *lactis* MG1363 with pMG36e	This study
*L. lactis* subsp. *lactis* MG1363/pMG36e-*bgl*1	*L. lactis* subsp. *lactis* MG1363 with pMG36e-*bgl*1	This study
*L. lactis* subsp. *lactis* MG1363/pMG36e-*cbh*2	*L. lactis* subsp. *lactis* MG1363 with pMG36e-*cbh*2	This study
*L. lactis* subsp. *lactis* MG1363/pMG36e-*egl*3	*L. lactis* subsp. *lactis* MG1363 with pMG36e-*egl*3	This study
*L. lactis* subsp. *lactis* MG1363/pMG36e-*usp*45-*bgl*1	*L. lactis* subsp. *lactis* MG1363 with pMG36e-*usp*45-*bgl*1	This study
*L. lactis* subsp. *lactis* MG1363/pMG36e-*usp*45-*cbh*2	*L. lactis* subsp. *lactis* MG1363 with pMG36e-*usp*45-*cbh*2	This study
*L. lactis* subsp. *lactis* MG1363/pMG36e-*usp*45-*egl*3	*L. lactis* subsp. *lactis* MG1363 with pMG36e-*usp*45-*egl*3	This study
Plasmids		
pMD18-T	Amp^r^	Takara Biotechnology Co., Ltd.
pMD18-T-*usp*45-*bgl*1	Amp^r^, clone *usp*45-*bgl*1 fusion gene	This study
pMD18-T-*usp*45-*cbh*2	Amp^r^, clone *usp*45-*cbh*2 fusion gene	This study
pMD18-T-*usp*45-*egl*3	Amp^r^, clone *usp*45-*egl*3 fusion gene	This study
pMG36e	Em^r^; expression vector with the P32 promoter, multiple cloning sites (MCF) and prtP translational terminator	Liu et al. [20]
pMG36e-*bgl*1	Em^r^; expression of *bgl*1	This study
pMG36e-*cbh*2	Em^r^; expression of *cbh*2	This study
pMG36e-*egl*3	Em^r^; expression of *egl*3	This study
pMG36e-*usp*45-*bgl*1	Em^r^, secretory expression of *bgl*1	This study
pMG36e-*usp*45-*cbh*2	Em^r^, secretory expression of *cbh*2	This study
pMG36e-*usp*45-*egl*3	Em^r^, secretory expression of *egl*3	This study

### Construction and examination of fusion genes

RNA was extracted from *T. reesei* 3.3711 with an E.Z.N.A.™ Fungal RNA Kit (Omega Bio-tek, Inc., Shanghai, China), and cDNA was obtained using a First-Strand cDNA Synthesis Kit (Omega Bio-tek, Inc., Shanghai, China). Primer P1 with *Eco*RI (5′-TTGAATTCCGTTGTACCTCCTGCAGGGACTC-3′) and primer P2 with *Bam*HI (5′-TTCGGATCCGCTACCGACAGAGTGCTCGTCAG-3′) were designed according to the *bgl1* gene sequence (GenBank Accession No. U09580.1) and were used to amplify the β-glucosidase catalytic domains of the *bgl*1 gene without the signal peptide and intron fragments (2139 bp). Polymerase chain reaction (PCR) was performed with a total volume of 25 μL containing 12.5 μL of 2× PrimeSTAR Max DNA polymerase (TakaRa Biotechnology Co., Ltd., Dalian, China), 1 μL cDNA and 1 μL of each primer. The PCR program was as follows: pre-denaturation at 94 °C for 5 min, followed by 30 cycles consisting of denaturation at 98 °C for 15 s, annealing at 55 °C for 15 s, extension at 72 °C for 4 min, and final elongation at 72 °C for 10 min. The fusion gene, *usp*45-*egl*3, was constructed with the same methods described in our previous study [20]. The correct *bgl*1 gene fragment was linked with *usp*45 (81 bp) of HT1 using the overlapping extension PCR method, and the restriction enzyme sequences were removed by a company (Nanjing Genebay Biotech Co., Ltd., Nanjing, China). The CD region of the *cbh*2 gene (GenBank Accession No. M55080.1) without a fraction of its signal peptide was also linked with *usp*45 (81 bp) of HT1 using the overlapping extension PCR method from a company (Shanghai Generay Biotech Co., Ltd., Shanghai, China). The fusion genes *usp*45-*bgl*1, *usp*45-*cbh*2, and *usp*45-*egl*3 were cloned into pMD18-T and sequenced. The sequences of the fusion genes were BLAST searched with reference genes in NCBI.

### Construction and transformation of recombinant plasmid

The corrected pMD18-T vector containing fusion genes and pMG36e were digested with the appropriate restriction enzymes in our laboratory. The fusion gene fragments and the digested pMG36e were linked via T4-DNA ligase (TakaRa Biotechnology Co., Ltd. Dalian, China) to produce the recombinant vectors pMG36e-*usp*45-*bgl*1, pMG36e-*usp*45-*cbh*2, and pMG36e-*usp*45-*egl*3. Then, the recombinant vectors were introduced into DH5α using a standard CaCl_2_ transformation method [25], and transformed into HT1 via electroporation using an Eppendorf multiporator (Eppendorf AG, Hamburg, Germany) [26], at a pulse voltage of 2.15 kV and a pulse time of 4.8 ms.

### Screening and identification of positive clones

Positive DH5α*/*pMD18-T-*usp*45-*bgl*1, DH5α*/*pMD18-T-*usp*45-*cbh2,* and DH5α*/*pMD18-T-*usp*45-*egl*3 transformants were screened via the white colour of the clones. Positive DH5α*/*pMG36e-*usp*45-*bgl*1, DH5α*/*pMG36e-*usp*45-*cbh*2, DH5α*/*pMG36e-*usp*45-*egl*3, HT1*/*pMG36e-*usp*45-*bgl*1, HT1*/*pMG36e-*usp*45-*cbh*2 and HT1*/*pMG36e-*usp*45-*egl*3 transformants were screened via erythromycin selection. pMD18-T-*usp*45-*egl*3 and pMG36e-*usp*45-*egl*3 were identified via the *Sma* I and *Xba* I digestion method. pMD18-T-*usp*45-*cbh2*, pMG36e-*usp*45-*cbh*2, pMD18-T-*usp*45-*bgl*1 and pMG36e-*usp*45-*bgl*1 were identified via the *Sma* I and *Sph* I digestion method. After digestion, the low-molecular-weight fragment was extracted using a Gel Extraction Mini Kit (Omega Bio-tek, Inc., Shanghai, China), which was sequenced and BLAST searched with reference genes in NCBI.

GM17 plates containing 5 g/L of carboxymethyl cellulose (CMC) and 5 μg/mL of erythromycin (pH 7.0) were used to screen the positive HT1*/*pMG36e-*usp*45-*bgl*1, HT1*/*pMG36e-*usp*45-*cbh*2 and HT1*/*pMG36e-*usp*45-*egl*3 recombinants after culturing for 48 h at 30 °C. The plates were then exposed to 1 g/L Congo red solution. After incubation for 30 min, the plates were washed with 1 mol/L NaCl to reveal the clear zones against a red background that developed via hydrolysis of CMC. The plates were rinsed with 5 g/L acetic acid to maximally delineate the zones of clearing.

### Enzyme assays and protein analysis of recombinant *L. lactis*

According to the procedure in Biofuels: Methods and Protocols written by Mielenz [27], the maximum secretory activities of HT1*/*pMG36e-*usp*45-*bgl*1, HT1*/*pMG36e-*usp*45-*cbh*2, and HT1*/*pMG36e-*usp*45-*egl*3 were individually detected using cellobiose, avicel and CMC. The filter paper activity of the recombinants when combined was detected using Whatman No. 1 filter paper as the substrate. One unit of activity was defined as the amount of enzyme that produced 1 μmol of reducing sugar per minute in glucose equivalents and the enzyme activity was assayed in triplicate. The molecular masses of intracellular and extracellular enzymes of recombinant HT1*/*pMG36e-*usp*45-*bgl*1, HT1*/*pMG36e-*usp*45-*cbh*2 and HT1*/*pMG36e-*usp*45-*egl*3 were estimated via sodium dodecyl sulphate–polyacrylamide gel electrophoresis (SDS-PAGE) as described in our previous study [20].

### Recombinants ensiling alfalfa

Alfalfa was planted on September 20, 2015, in ten different fields (humid subtropical climate, latitude 32°01′59.81″N, longitude 118°50′13.63″E, altitude above sea level 17 m) of Nanjing Agricultural University (Nanjing, China). The area of each field was 100 m^2^. The alfalfa was at the early flowering stage on May 11, 2016, and was harvested for making silage, immediately. Fresh alfalfa was chopped into 1- to 2-cm-long pieces by a forage chopper (Sh-2000, Shanghai Donxe Industrial Co., Ltd., Shanghai, China). Prior to ensiling, the alfalfa had a DM of 221 g/kg fresh matter, a pH value of 6.28 and a buffering capacity of 244 mEq/kg DM. The composition of structural carbohydrates was 441 g for neutral detergent fibre (NDF)/kg DM, 326 g for acid detergent fibre (ADF)/kg DM, and 84.3 g/kg DM for acid detergent lignin (ADL). The total WSC content was 52.82 g/kg DM, while the fraction of the individual soluble carbohydrates (g/kg DM) was as follows: glucose (5.81), fructose (5.04), and xylose (5.68). The epiphytic lactic acid bacteria (LAB, 3.93 lg cfu/g FM) on the alfalfa was less than aerobic bacteria (7.36 lg cfu/g FM) and yeast (4.98 lg cfu/g FM) and the fermentation efficiency (23.8) was lower than 35.

The strains HT1, HT1*/*pMG36e-*usp*45-*bgl*1, HT1*/*pMG36e-*usp*45-*cbh*2 and HT1*/*pMG36e-*usp*45-*egl*3 were used after being cultured in GM17 broth at 30 °C for 30 h. HT2 consisted of HT1*/*pMG36e-*usp*45-*bgl*1, HT1*/*pMG36e-*usp*45-*cbh*2 and HT1*/*pMG36e-*usp*45-*egl*3 at an equal ratio (1:1:1). EN derived from *Trichoderma reesei* was purchased from a company (Rueyang Biotechnology Co., Ltd, Wuxi, China). The activity of EN was measured: cellulase 50,000 U/g.

Alfalfa obtained from 8 random fields was mixed into a pile and then was separated into 80 piles in the laboratory. The 80 piles (720 g per pile) were randomly ensiled with 5 additive treatments (without additive as the Control, and with EN, HT1, HT1 + EN or HT2). The 16 piles per additive treatment were separately filled into 16 experimental silos (polyvinyl chloride bottle, 1 L) immediately. According to McFarland turbidity standards, adjusted HT1 and HT2 were separately added to make the inoculation 1 × 10^6^ colony-forming units (cfu)/g fresh matter (FM). EN was added at 2 g/kg of FM. EN was mixed with adjusted HT1 as HT1 + EN (dose at 2 g/kg EN + 1 × 10^6^ cfu/g HT1 of FM). The control was sprayed with the same amount of distilled water alone. The 80 silos were sealed using the same method described in the report of Liu et al. [28]. Four silos of each additive treatment were randomly selected and opened after ensiling at ambient temperature (23–37 °C) for 1, 6, 18 and 60 days.

### Microbiological and chemical analysis

LAB, aerobic bacteria and yeast were counted according to the method described by Liu et al. [28]. Clostridial spores were enumerated by surface plating on supplemented Reinforced Clostridium Agar (Product Code: HB0316; Hopebio Co., Ltd., Qingdao, China) and anaerobic incubation for 3 days at 37 °C according to the method described by Jonsson [29]. Fifty grams of alfalfa material was mixed with 200 mL of distilled water, and stored at 4 °C for 18 h. The mixture was then filtered, and the filtrate was used to determine pH value. The DM and WSC content and buffer capacity of alfalfa material were measured using the method described by Liu et al. [18]. Ground dried samples were used to determine monosaccharide contents (glucose, fructose, and xylose). Sugars in alfalfa were extracted with 80% ethanol and measured by Agilent HPLC 1260 equipped with a column (Skim-pack SCR-101C, Shimadzu, Inc. Japan) and refractive index detector [30]. The analytical column was performed at 85 °C using HPLC grade water as the mobile phase, with a flow rate of 1 mL/min. The fermentation coefficient (FC) of alfalfa silage was predicted according to the formula of Addah et al. [31], as follows: FC = DM % + 8 × WSC g/kg DM ÷ BC mEq/kg DM, where BC is the buffering capacity of the fresh alfalfa. FC expresses whether the fresh forage will ensile easily or will be difficult to ensile (FC > 45 = easy, FC < 35 = difficult to ensile). The crude protein of alfalfa was analysed using the methods in AOAC-984.13 [32]. The contents of NDF and ADF of alfalfa material were measured using the method of Mertens et al. [33] and AOAC-973.18 [34], respectively. After silos were opened, the DM, WSC, sugars, crude protein, NDF, and ADF of the silage were measured by the same method used for the alfalfa material. The DM loss of the silage was estimated by measuring the differences in DM weights in the same silo after the silo was sealed for 2 h and after ensiling for 60 days. Corrected DM was calculated according to the formula of Porter et al. [35]. DM recovery was calculated by the formula: DM recovery % = (100 − DM loss) %. The filtrate was treated using the same approach as the alfalfa material and was used to measure the pH value, ammonia-N and organic acid content as in our previous study [28]. Fermentation quality was assessed by V-score using the same method in our previous study [18].

### Data statistics and analyses

An exponential decay model was used to fit the WSC and monosaccharide (glucose, fructose, and xylose) data using IBM Statistical Packages for the Social Sciences (IBM SPSS 20.0 for Windows) to describe the sugar change characteristics with the following equation:

1 $$y\, = \,y0\, + \,a*\exp \left( { - b*x} \right)$$

where *y* (g/kg DM) is the residue at any time *x* (day), *y*0 (g/kg DM) is the total residual fraction after 60 days of ensiling, *a* (g/kg DM) is the consumable fraction, *b* (day^−1^) is the fractional consumption rate of *a* and *x* is the ensilage time (day) [4].

The statistical analyses were performed using the IBM Statistical Packages for the Social Sciences (IBM SPSS 20.0 for Windows). The data were analysed by two-way analysis of variance (ANOVA, general linear models) (five treatments × four ensiling time × four replicates) to evaluate the effects of additives, ensiling time and their interaction on the fermentation characteristics, structural carbohydrate degradability and nonstructural carbohydrate fermentation of alfalfa silages. The data were analysed by one-way ANOVA (five treatments × four replicates) to evaluate the effects of additives on DM, DM recovery, CP and microbial composition of alfalfa silages after ensiling for 60 days. The means were then compared for significance using Tukey’s test at *P* < 0.05.

## Results

### Identification and enzyme expression level of recombinants

**Fig. 2:**
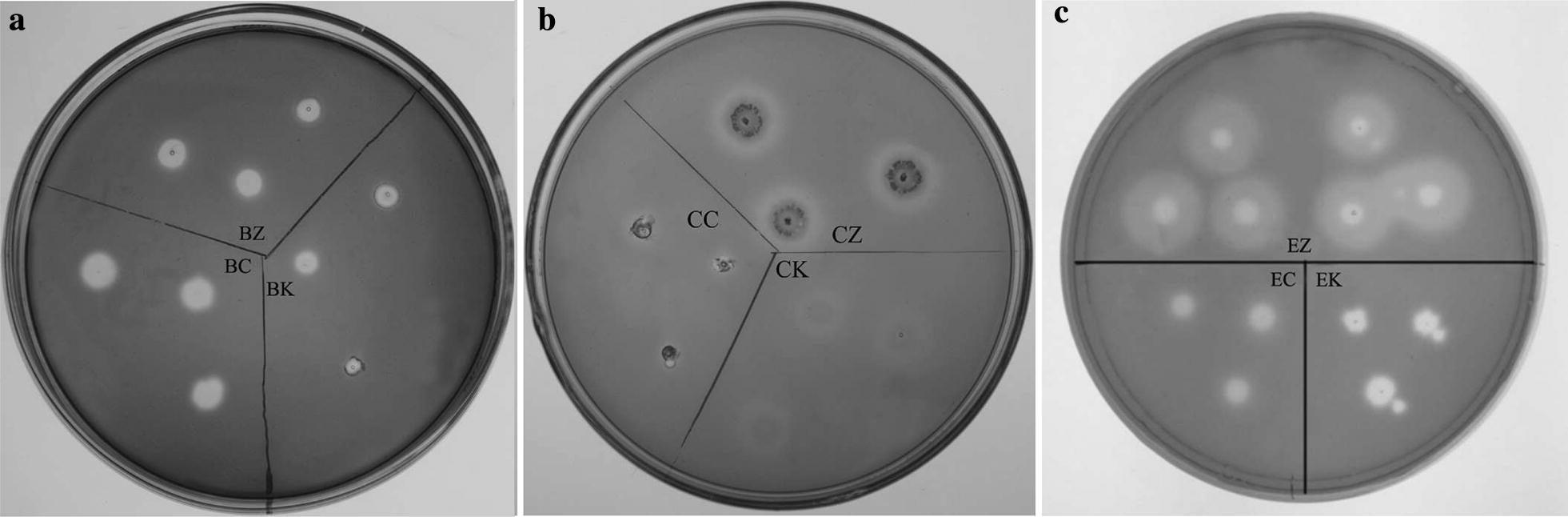
Effects of recombinants expressing β-glucosidase, cellobiohydrolase and endoglucanase on the media containing sodium carboxymethylcellulose. **a** BC, *L. lactis* subsp. lactis MG1363/pMG36e; BK, *L. lactis* subsp. *lactis* MG1363/pMG36e-*egl*3; BZ, *L. lactis* subsp. *lactis* MG1363/pMG36e-*usp*45-*egl*3. **b** CC, *L. lactis* subsp. lactis MG1363/pMG36e; CK, *L. lactis* subsp. *lactis* MG1363/pMG36e-*egl*3; CZ, *L. lactis* subsp. *lactis* MG1363/pMG36e-*usp*45-*egl*3. **c** EC, *L. lactis* subsp. lactis MG1363/pMG36e; EK, *L. lactis* subsp. *lactis* MG1363/pMG36e-*egl*3; EZ, *L. lactis* subsp. *lactis* MG1363/pMG36e-*usp*45-*egl*3

**Fig. 3:**
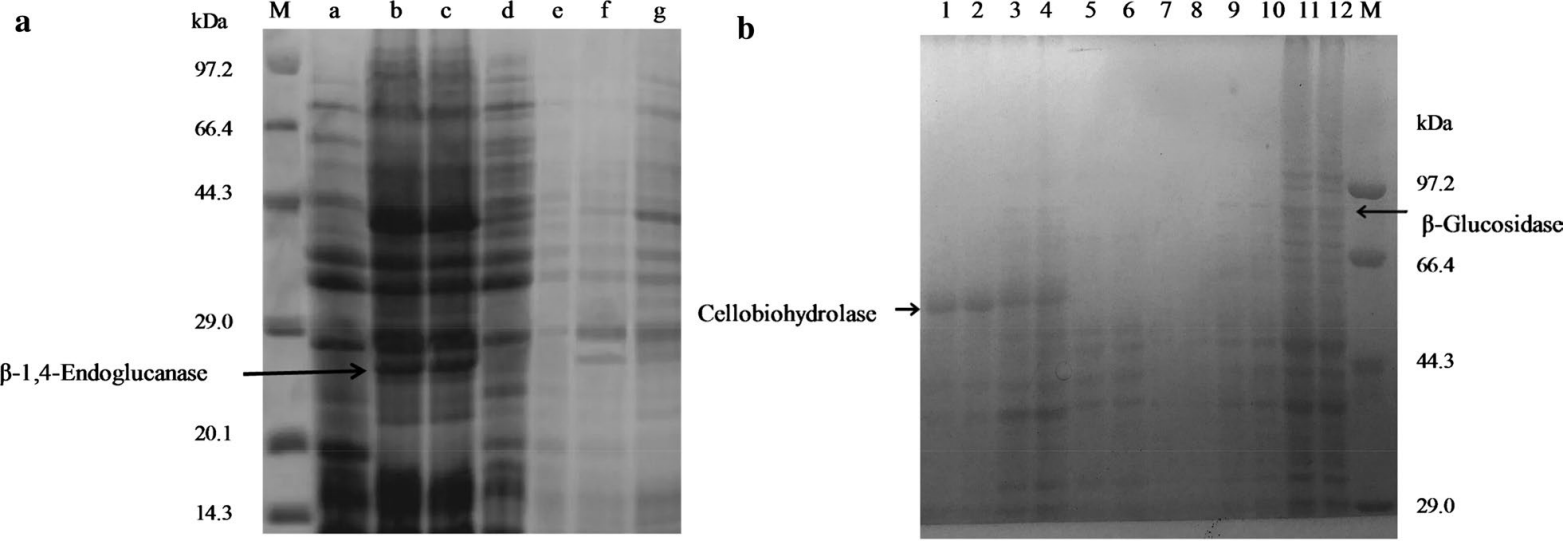
SDS-PAGE of the recombinant strains. **a** M, protein molecular weight marker; lane a, intracellular protein of *L. lactis* subsp. *lactis* MG1363; lanes b and c, intracellular protein of *L. lactis* subsp. *lactis* MG1363/pMG36e-*usp*45-*egl*3; lane d, intracellular protein of *L. lactis* subsp. *lactis* MG1363/pMG36e; lane e, extracellular protein of *L. lactis* subsp. *lactis* MG1363/pMG36e; lanes f and g, extracellular protein of *L. lactis* subsp. *lactis* MG1363/pMG36e-*usp*45-*egl*3; **b** lanes 1 and 2, extracellular protein of *L. lactis* subsp. *lactis* MG1363/pMG36e-*usp*45-*cbh*2; lanes 3 and 4, intracellular protein of *L. lactis* subsp. *lactis* MG1363/pMG36e-*usp*45-*cbh*2; lanes 5 and 6, intracellular protein of *L. lactis* subsp. *lactis* MG1363/pMG36e; lanes 7 and 8, extracellular protein of *L. lactis* subsp. *lactis* MG1363/pMG36e; lanes 9 and 10, extracellular protein of *L. lactis* subsp. *lactis* MG1363/pMG36e-*usp*45-*bgl*1; lanes 11 and 12, intracellular protein of *L. lactis* subsp. *lactis* MG1363/pMG36e-*usp*45-*bgl*1; M, protein molecular weight marker

**Fig. 4:**
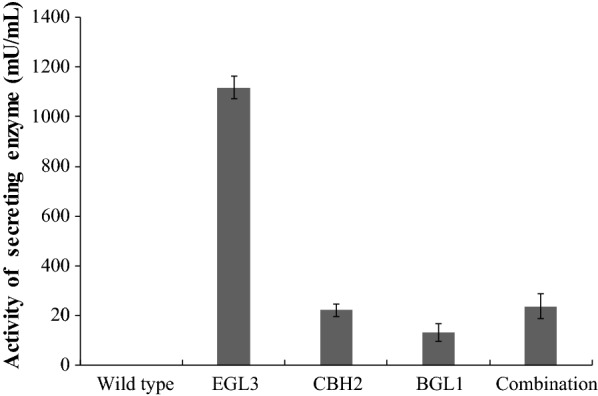
The activities of secreting enzyme in each recombinant and their combination on degrading paper. HT1, wild-type *L. lactis* subsp. *lactis* MG1363; BGL1, HT1/pMG36e-*usp*45-*bgl*1; CBH2, HT1/pMG36e-*usp*45-*cbh*2; EGL3, HT1/pMG36e-*usp*45-*egl*3; combination, the mixed strains of BGL1, CBH2 and EGL3

Compared with the functional fragments of the reference genes (GenBank Accession No. M60178.1, U09580.1, M55080.1, and AB003694.1), there was no variation in the fusion genes *usp*45-*bgl*1, *usp*45-*cbh*2 and *usp*45-*egl*3, as exhibited by the 100% similarity to the sequence in GenBank. The positive transformants HT1*/*pMG36e-*usp*45-*bgl*1, HT1*/*pMG36e-*usp*45-*cbh*2 and HT1*/*pMG36e-*usp*45-*egl*3, degraded CMC, as demonstrated by the clear transparent zone, while HT1*/*pMG36e, HT1*/*pMG36e-*bgl*1, HT1*/*pMG36e-*cbh*2, and HT1/pMG36e-*egl*3 did not have clear transparent zones (Fig. 2). Furthermore, SDS-PAGE separately revealed an evident idio-strap of approximately 78 kDa in HT1*/*pMG36e-*usp*45-*bgl*1, 50 kDa in MG1363/pMG36e-*usp*45-*cbh2* and 25 kDa in HT1/pMG36e-*usp*45-*egl*3 after culturing for 28 h compared with HT1/pMG36e in both the supernatant and cell samples (Fig. 3). The present study showed that the maximum extracellular endoglucanase expression level was 1118 mU/mL in *L. lactis*, which was higher than the expression levels of extracellular cellobiohydrolase (222 mU/mL) and β-glucosidase (131 mU/mL) (Fig. 4). In addition, HT2 showed high filter paper activity (236 mU/mL), which indicated that the combined recombinant *L. lactis* had a potential role in degrading lignocellulose.

### Changes in fermentation parameters in silages after ensiling

The additives, ensiling time and their interaction significantly influenced pH, lactic acid, acetic acid, the ratio of lactic acid to acetic acid (LA/AA), butyric acid, ammonia-N and V-score (*P* < 0.05) (Table 2). The lactic acid content in the control silage increased to 41.1 g/kg DM during 18 days of ensiling but decreased to 16.3 g/kg DM at the final ensiling time (60 days), while the contents of acetic acid and ammonia-N in the control silage individually increased to 42.5 g/kg DM and 246 g/kg N, respectively, during 60 days of ensiling. Under this condition of low LA/AA, the pH in the control silage decreased to 4.39 during 18 days of ensiling but increased to 5.35 after ensiling for 60 days. In addition, high butyric acid content was found in the control silage (36.94 g/kg DM) after ensiling for 60 days, indicating poor quality (38.2 quality score). The HT1-treated silage had similar changes in pH, lactic acid, acetic acid and butyric acid with the control silage during 60 days of ensiling (*P* > 0.05). Therefore, the HT1-treated silage had a poor-quality score (36.5). Compared with the control, EN, HT1 + EN and HT2 had better effects on increasing lactic acid content and decreasing ammonia-N content at each ensiling time (*P* < 0.05 or *P* > 0.05). In addition, the EN-treated silage had higher acetic acid content (*P* < 0.05 or *P* > 0.05), and the HT1 + EN- and HT2-treated silages had lower acetic acid and ammonia-N content at each ensiling time (*P* < 0.05 or *P* > 0.05). Therefore, lower pH values in the EN-, HT1 + EN- and HT2-treated silages were observed compared to the control silage after ensiling for 60 days (*P* < 0.05), which eliminated butyric fermentation and improved the fermentation quality (quality score > 65). However, HT1 + EN- and HT2-treated silage had higher lactic acid content and LA/AA (*P* < 0.05) and lower contents of acetic acid (*P* < 0.05) and ammonia-N (*P* > 0.05) than EN-treated silage after ensiling for 60 days. Thus, the HT1 + EN- and HT2-treated silage obtained a higher quality score than the EN-treated silage (79.7 and 83.6 vs 68.8). No differences in the majority of fermentation quality parameters were found between HT1 + EN and HT2 at most of the ensiling times (*P* > 0.05).

**Table 2:** Fermentative characteristics of high-moisture alfalfa silages after ensiling

Items	Additives	Time (days)				SEM	Significance		
		1	6	18	60		Additives	Time	Additives × time
pH	Control	5.80^aA^	4.57^aB^	4.39^aB^	5.35^aA^	0.059	< 0.001	< 0.001	< 0.001
	EN	5.80^aA^	4.42^abC^	4.17^bcD^	4.62^bB^				
	HT1	4.95^cB^	4.42^abC^	4.32^abC^	5.22^aA^				
	HT1 + EN	5.23^bA^	4.40^abB^	4.29^abcC^	4.47^bB^				
	HT2	4.76^dA^	4.20^bC^	4.12^cC^	4.49^bB^				
Lactic acid (g/kg DM)	Control	8.67^bB^	40.1^bA^	41.1^bA^	16.3^bB^	2.914	< 0.001	< 0.001	< 0.001
	EN	10.3^bC^	45.2^abAB^	56.6^aA^	33.8^bB^				
	HT1	16.4^aC^	40.3^bB^	49.4^abA^	18.3^bC^				
	HT1 + EN	16.1^aC^	52.5^aB^	50.1^abB^	69.0^aA^				
	HT2	17.4^aD^	41.9^bC^	58.2^aB^	67.1^aA^				
Acetic acid (g/kg DM)	Control	12.0^aC^	22.8^abBC^	32.4^abAB^	42.5^bA^	3.027	< 0.001	< 0.001	< 0.001
	EN	12.2^aC^	24.8^aBC^	33.6^aB^	79.2^aA^				
	HT1	2.10^bC^	16.0^bcBC^	24.6^bB^	46.2^bA^				
	HT1 + EN	5.09^bD^	19.0^abC^	30.9^abB^	39.9^bA^				
	HT2	1.70^bD^	9.56^cC^	24.3^bB^	32.4^bA^				
Propionic acid (g/kg DM)	Control	0.19	0.00	0.00	5.69	1.028	0.150	0.011	0.110
	EN	0.00	0.00	0.00	0.00				
	HT1	0.00^B^	0.00^B^	0.00^B^	3.79^A^				
	HT1 + EN	0.00	0.00	0.00	0.00				
	HT2	0.00	0.00	0.00	0.00				
Butyric acid (g/kg DM)	Control	0.00^B^	0.00^B^	0.59^B^	36.94^aA^	2.700	< 0.001	< 0.001	< 0.001
	EN	0.00	0.00	0.00	0.00				
	HT1	0.00B	0.00B	0.00B	29.89^aA^				
	HT1 + EN	0.00	0.00	0.00	0.00^b^				
	HT2	0.00	0.00	0.00	0.00^b^				
Ammonia-N (g/kg N)	Control	50.2^aB^	103^aB^	113^aB^	246^aA^	11.49	< 0.001	< 0.001	< 0.001
	EN	48.2^abC^	79.2^bB^	87.1^bB^	129^bA^				
	HT1	35.6^bcB^	65.8^bcB^	83.26^bB^	210^abA^				
	HT1 + EN	37.7^abcD^	64.7^bcC^	91.0^bB^	108^bA^				
	HT2	34.0^cD^	51.9^cC^	81.1^bB^	106^bA^				
LA/AA	Control	0.74 ^dBC^	1.77^bA^	1.26^cAB^	0.38^bC^	0.254	< 0.001	< 0.001	< 0.001
	EN	0.84^dB^	1.83^bA^	1.68^bcAB^	0.44^bC^				
	HT1	7.84^bA^	2.64^bB^	2.04^abBC^	0.43^bC^				
	HT1 + EN	3.16^cA^	2.81^bA^	1.65^bcB^	1.75^aB^				
	HT2	10.33 aA	4.40 aB	2.39 aC	2.08^aC^				
V-score	Control	99.6^abA^	85.9^cA^	79.8^bA^	38.2^bcB^	2.519	< 0.001	< 0.001	< 0.001
	EN	99.5^bA^	91.7^bB^	87.9^aB^	68.8^abC^				
	HT1	100^aA^	95.6^abAB^	88.8^aB^	36.5^cC^				
	HT1 + EN	100^aA^	95.4^abA^	88.3^aB^	79.7^aC^				
	HT2	100^aA^	99.5^aA^	91.7^aB^	83.6^aC^				

Values with different superscript lowercase letters show significant differences among treatments in the same ensiling day, values with different superscript capital letters show significant differences among ensiling days in the same treatment (*P* < 0.05) according to Tukey’s test

Control, silage treated without additives; DM, dry matter; EN, cellulase; HT1, wild-type *L. lactis* subsp. *lactis* MG1363; HT1 + EN, combination of HT1 and EN; HT2, combination of transgenically engineered *L. lactis* strains HT1*/*pMG36e-*usp*45-*bgl*1, HT1*/*pMG36e-*usp*45-*cbh*2, and HT1*/*pMG36e-*usp*45-*egl*3; N, nitrogen; LA/AA, ratio of lactic acid to acetic acid; SEM, standard error of the means

### Changes in structural carbohydrates in silages during ensiling

**Fig. 5:**
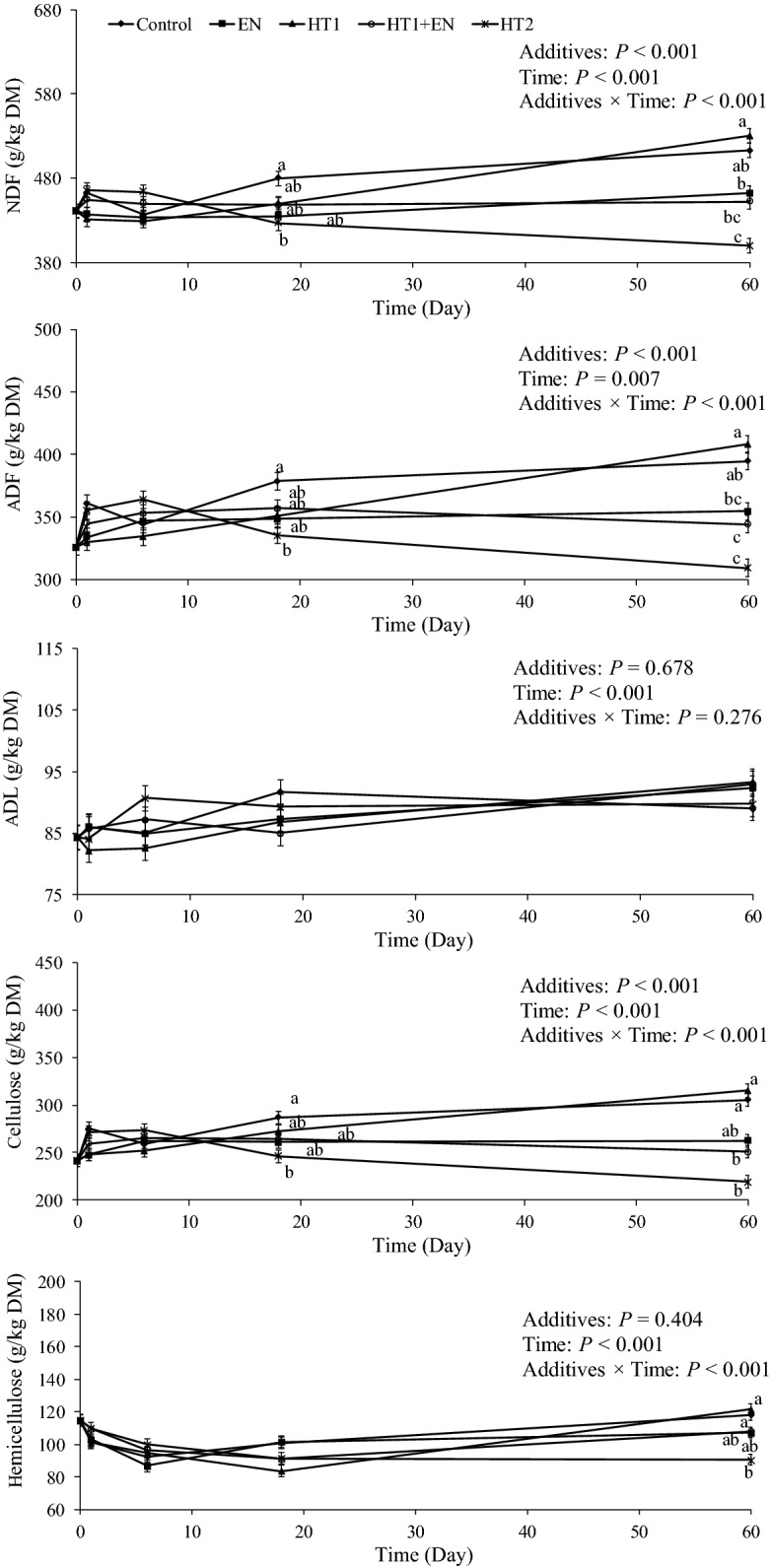
Changes in NDF, ADF, ADL, cellulose and hemicellulose in alfalfa silages during ensiling for 60 days. Different lowercase letters (a–c) indicated difference at *P* < 0.05 among additive treatments on the same ensiling day. Control, silage treated without additives; EN, cellulase; HT1, wild-type *L. lactis* subsp. *lactis* MG1363; HT1 + EN, combination of HT1 and EN; HT2, combination of transgenically engineered *L. lactis* strains HT1*/*pMG36e-*usp*45-*bgl*1, HT1*/*pMG36e-*usp*45-*cbh*2, and HT1*/*pMG36e-*usp*45-*egl*3

The interaction of additive and ensiling time had a significant effect on most of the structural carbohydrates of alfalfa silages (*P* < 0.05), and ensiling time significantly influenced ADL (*P* < 0.05) (Fig. 5). All structural carbohydrates in the HT1-treated and control silages showed an increasing tendency during 60 days of ensiling. No differences in NDF, ADF, cellulose, hemicellulose and ADL were found between the control and additive treatments after ensiling for 1 day and 6 days (*P* > 0.05). In contrast, after ensiling for 18 and 60 days, compared with the control silages, the EN-, HT1 + EN- and HT2-treated silages had lower contents of NDF, ADF and cellulose (*P* < 0.05 or *P* > 0.05). Furthermore, the HT2-treated silage had the lowest contents of NDF, ADF, cellulose and hemicellulose, followed by the HT1 + EN- and EN-treated silages after ensiling for 18 and 60 days (*P* > 0.05).

### Changes in nonstructural carbohydrates in silages during ensiling

**Fig. 6:**
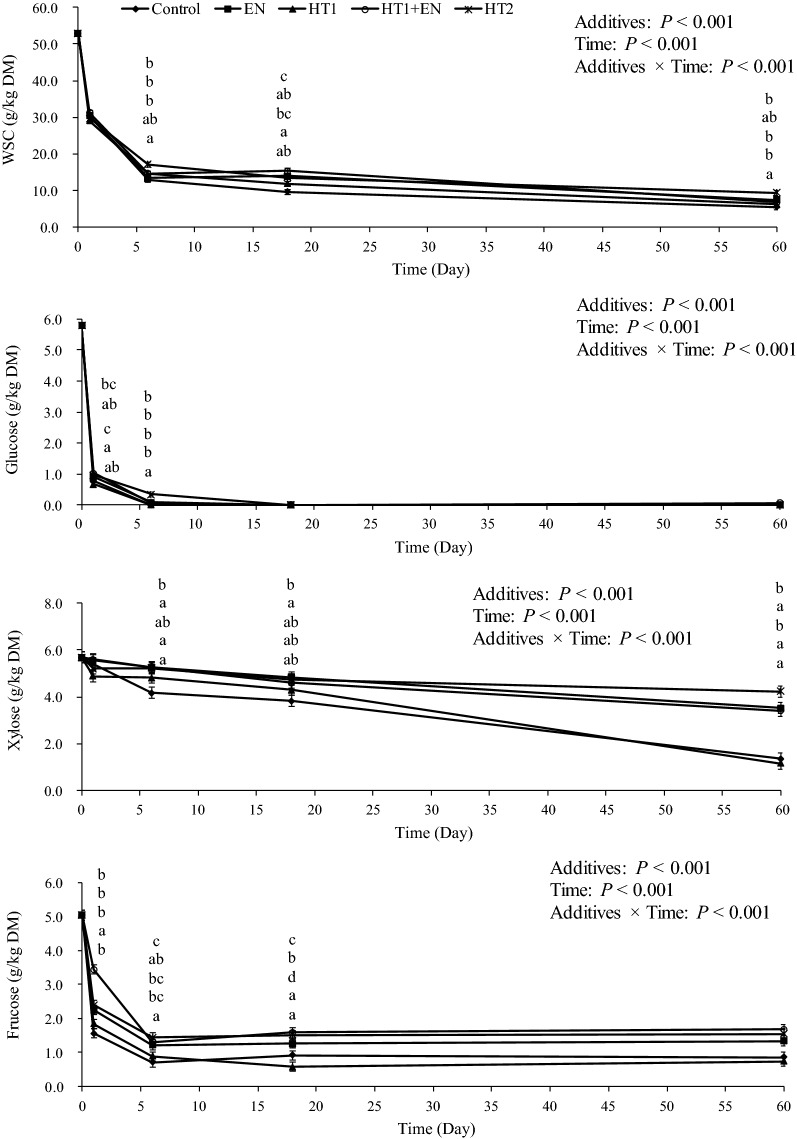
Changes in WSC, glucose, xylose and fructose in alfalfa silages during ensiling for 60 days. Different lowercase letters (a–d, from top to bottom orderly corresponding to additive treatments) indicated difference at *P* < 0.05 among additive treatments on the same ensiling day. WSC, water-soluble carbohydrates. Control, silage treated without additives; EN, cellulase; HT1, wild-type *L. lactis* subsp. *lactis* MG1363; HT1 + EN, combination of HT1 and EN; HT2, combination of transgenically engineered *L. lactis* strains HT1*/*pMG36e-*usp*45-*bgl*1, HT1*/*pMG36e-*usp*45-*cbh*2, and HT1*/*pMG36e-*usp*45-*egl*3

Additives, ensiling time and their interaction significantly influenced WSC and glucose, fructose and xylose (*P* < 0.05) (Fig. 6). As the ensiling time was prolonged to 60 days, nonstructural carbohydrates decreased (*P* < 0.05); in particular, glucose disappeared in all silages after ensiling for 18 days. After ensiling for 1 day, the HT1 + EN-treated silage had higher glucose than the control and the HT1-treated silage (*P* < 0.05) and had the highest fructose content (*P* < 0.05). However, EN, HT1 + EN and HT2 increased the residual WSC, glucose, fructose, and xylose in silage compared with the control after ensiling for 6, 18 and 60 days (*P* < 0.05 or *P *> 0.05). Lower xylose content was found in the HT1-treated and control silages compared with the EN-, HT1 + EN- and HT2-treated silages after ensiling for 60 days (*P* < 0.05). Furthermore, the HT2-treated silage had the highest WSC, xylose and fructose contents, followed by the HT1 + EN- and EN- treated silages (*P *> 0.05).

### Kinetic analysis of nonstructural carbohydrates in silages during ensiling

The present study used a first-order exponential decay model to fit the measured data of nonstructural carbohydrates, and the kinetic parameters are shown in Table 3. Only WSC, glucose and fructose (*R*^2^ from 0.941 to 1) were suitable to fit the model, since the correlation coefficients (*R*^2^ values) of the tested data approached 1. The additives significantly influenced parameters of WSC, glucose and fructose (*P* < 0.05), except for the *b* value in glucose (*P* = 0.054).

**Table 3:** Kinetic parameters of nonstructural carbohydrates reduction in alfalfa silages based on first-order exponential decay model *y* = *y*0 + a*e^(−b*x)^

Additives	WSC			Glucose			Fructose		
	*y*0	*a*	*b*	*y*0	*a*	*b*	*y*0	*a*	*b*
Control	8.98^e^	43.6^a^	0.69^c^	0.00^b^	5.81^a^	1.99	0.84^c^	4.20^b^	1.67^a^
EN	11.4^c^	41.3^c^	0.77^b^	0.05^b^	5.76^a^	1.77	1.28^b^	3.76^c^	1.33^b^
HT1	10.2^d^	42.3^b^	0.77^b^	0.00^b^	5.81^a^	2.00	0.73^d^	4.31^a^	1.35^b^
HT1 + EN	12.0^b^	40.6^d^	0.73^bc^	0.04^b^	5.77^a^	1.75	1.50^a^	3.56^d^	0.65^c^
HT2	13.2^a^	39.5^e^	0.85^a^	0.13^a^	5.64^b^	1.70	1.51^a^	3.53^d^	1.35^b^
SEM	0.014	0.125	0.017	0.015	0.035	0.108	0.010	0.009	0.040
Significance	< 0.001	< 0.001	< 0.001	< 0.001	0.004	0.054	< 0.001	< 0.001	< 0.001

Means within a symbol with different superscript lowercase letters (a–e) differ (*P* < 0.05) according to Tukey’s test

According to Li et al. [28], first-order exponential decay model was *y* = *y*0 + *ae* ^(−b*x)^: *y* (g/kg DM) is the residue at any time *x* (day); *y*0 (g/kg DM) is the total residual fraction after 60 days of ensiling; *a* (g/kg DM) is the consumable fraction; *b* (day^−1^) is the fractional consumption rate of *a* and *x* is the ensilage time (day)

EN, cellulase; HT1, wild-type *L. lactis* subsp. *lactis* MG1363; HT1 + EN, combination of HT1 and EN; HT2, combination of transgenically engineered *L. lactis* strains HT1*/*pMG36e-*usp*45-*bgl*1, HT1*/*pMG36e-*usp*45-*cbh*2, and HT1*/*pMG36e-*usp*45-*egl*3; SEM, standard error of the means

The *y0* values for WSC, glucose and fructose in the HT2-treated silage were the highest (*P* < 0.05 or *P* > 0.05), indicating high residual sugars in the HT2-treated silage, while the *y0* values for WSC, glucose and fructose in the control silage were the lowest when compared with others (*P* < 0.05 or *P* > 0.05). The HT2-treated silage had the lowest *a* value for WSC, glucose and fructose when compared with others (*P* < 0.05 or *P* > 0.05), indicated by the low consumable sugar fraction. The *b* values for fructose in the EN-, HT1 + EN- and HT2-treated silages were lower than the control (*P* < 0.05), while the *b* values for WSC in the EN-, HT1 + EN- and HT2-treated silages were higher than in the control (*P *< 0.05). Furthermore, the *b* values for WSC and fructose in the HT1 + EN-treated silage were lower than those in the HT2-treated silage (*P* < 0.05), as indicated by the lower sugar consumption rate in HT2-treated silage.

### DM, DM recovery, crude protein and microbial composition of alfalfa silage after ensiling for 60 days

**Fig. 7:**
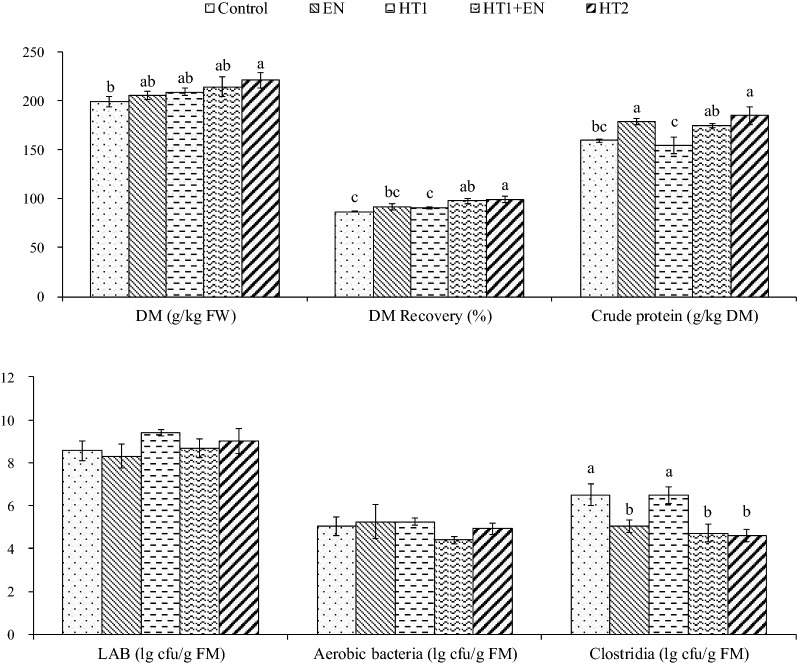
DM, DM recovery, crude protein and microbial composition of alfalfa silage after ensiling for 60 days. Means within a column with different letters (a–c) differ (*P* < 0.05) according to Tukey’s test. Control, silage treated without additives; EN, cellulase; HT1, wild-type *L. lactis* subsp. *lactis* MG1363; HT1 + EN, combination of HT1 and EN; HT2, combination of transgenically engineered *L. lactis* strains HT1*/*pMG36e-*usp*45-*bgl*1, HT1*/*pMG36e-*usp*45-*cbh*2, and HT1*/*pMG36e-*usp*45-*egl*3

After ensiling for 60 days, additives markedly influenced DM, DM recovery, crude protein, and clostridium number (*P* < 0.05) (Fig. 7). The highest DM recovery, contents of DM and crude protein were found in the HT2-treated silage, followed by HT1 + EN and EN (*P* > 0.05). In addition, compared with the control and HT1-treated silage, lower *Clostridia* spores were observed in HT2, HT1 + EN, and EN (*P* < 0.05).

### The effects of additives on the appearance evaluation of alfalfa silages

After ensiling for 60 days, silages showed different shapes, colours, smells, and textures. The HT1 and control silage emitted an unpleasant odour and appeared black and sticky. There were no clear leaves in the HT1-treated and control silages. In contrast, the EN-, HT1 + EN-, and HT2-treated silages emitted a fragrance and were yellow but not sticky. The leaves were easily distinguished in the EN-, HT1 + EN-, and HT2-treated silages, indicating good process (see Additional file 1).

## Discussion

pMG36e is known to be a constitutive expression vector for the inserted gene in *L. lactis* [36]. However, some reports have shown that the expression effect of some heterogeneous proteins appeared unstable [23, 37, 38], because the constitutive expression was correlated with the solubility of the heterogeneous protein [21]. In the present study, the active endoglucanase, cellobiohydrolase, and β-glucosidase of recombinant *L. lactis* were separately secreted into the culture medium, as indicated by correct folding and significant solubility.

The highest activities of the extracellular endoglucanase, cellobiohydrolase, and β-glucosidase in recombinant *L. lactis* were different, which demonstrated that the heterogeneous protein size and conformation were factors for heterologous protein secretion in *L. lactis* using *usp*45 [21, 22]. In addition, the introduction of the large heterogeneous gene results in an oversized plasmid that increases metabolic load and accelerates cell death [39]. This might be the reason that the *bgl*1, *cbh*2, and *egl*3 genes were unable to be expressed together in *L. lactis* using *usp*45 and pMG36e (unpublished data). Therefore, we had to separately express the *bgl*1, *cbh*2 and *egl*3 genes using *usp*45 and pMG36e in *L. lactis*. Furthermore, after deleting the inherent sequence of the signal peptide in *bgl*1, *cbh*2 and *egl*3 and using *usp*45, an enhancement in secretion was promoted. One study concluded that signal peptide and propeptide optimization could enhance secretion to increase heterologous protein production in *L. lactis* [21]. Additionally, the CMC activity of HT1/pMG36e-*usp*45-*egl*3 was higher than that of *Clostridium thermocellum* (1118 vs 140 mU/mL) and *Bacillus subtilis* J12 (1118 vs 165 mU/mL) [40, 41]. HT2 had higher filter paper activity than the average activity of cellulolytic rumen *Enterococcus* strains (236 vs 212 mU/mL) and *T. reesei* (236 *vs* 190 mU/mL) [4, 42]. These results indicated that the engineered *L. lactis* strains had stronger lignocellulose activities. The above outcomes could be attributed to the strategy for forming active cellulolytic proteins: (i) the intron and inherent sequence of the signal peptide in the *bgl*1, *cbh*2 and *egl*3 genes were deleted; and (ii) the sequences of the coding sequence in the *bgl*1, *cbh*2 and *egl*3 genes were separately fused with *usp*45.

The present study showed that lactic fermentation was not vigorous in the control silage after ensiling for 18 days, and as a result, insufficient lactic acid could not effectively decrease pH to prevent the emergence of *Clostridia* in the control silage. With an extended ensiling time of 60 days, vigorous butyric fermentation occurred, indicated by the poor fermentation quality of the control silage. This outcome could be caused by the low fermentation coefficient in high-moisture alfalfa, caused by low DM and WSC contents and high buffer capacity. Reports have concluded that high-moisture alfalfa is not easily fermented by epiphytic LAB, because it has low contents of DM and WSC and high buffer capacity [43, 44]. Similar to our previous studies [28], butyric fermentation led to high DM loss (low DM recovery), mainly in nutrient loss in the control silage. Markedly, protein degradation, considerable consumption of WSC and monosaccharides (glucose, fructose, and xylose), accumulation of ammonia-N and a sticky texture were observed in the control silage after ensiling for 60 days. Undoubtedly, many cell wall polysaccharides remained in the control silage. It has been suggested that measures should be used to well preserve high-moisture alfalfa well in the rainy season.

Cellulase has been used in ensiling bioprocesses because it degrades lignocellulose to provide sugars for LAB fermentation [9, 10]. In the present study, the curves of structural carbohydrate degradation, WSC and monosaccharide consumption demonstrated that EN played a role in degrading lignocellulose to improve the fermentation quality of silage, which was the same as the outcomes in our previous studies [17, 18]. However, the EN-treated silage did not exhibit vigorous lactic fermentation instead of acetic fermentation after ensiling for 60 days. These outcomes were explained by the high residual xylose derived from the degradation of lignocellulose, which stimulated heterolactic fermentation of the epiphytic LAB of alfalfa during the final ensiling period. A similar result was reported by Lynch et al. who found acetic fermentation in cellulase-treated alfalfa silages after ensiling for 70 days, since the acetic acid content was close to or surpassed the lactic acid content [12].

Many researchers have focused on improving fermentation quality by inoculation with LAB [18, 45]. The present study showed that HT1 promoted lactic fermentation during the first 18 days of ensiling, but did not eliminate butyric acid after ensiling for 60 days. This might be attributed to insufficient fermentative sugars for HT1 to produce lactic acid and restrain clostridial fermentation during a prolonged storage period. Similar to the control silage, the nutrient loss and cell wall polysaccharides increased in the HT1-treated silage. In addition, the sticky texture and unpleasant odour of the HT1-treated silage suggested that *L. lactis* could not ensile high-moisture alfalfa well in the rainy season. Kung reported that homofermentative LAB might lower silage pH relative to that in untreated silage, but the degree of reduction may or may not be sufficient to prevent clostridial growth, depending on the circumstances [46]. In the present study, low contents of DM and WSC and high buffer capacity in the ensiling material seriously limited the ability of HT1 to restrain clostridial fermentation.

Similar to previous studies [11, 17, 47], HT1 + EN promoted lactic fermentation compared with untreated high-moisture alfalfa silage after ensiling for 18 and 60 days. This resulted from the dual function of HT1 + EN: (i) cellulase derived from *T. reesei* degraded lignocellulose into sugars well; and (ii) HT1 fermented sugars via homolactic fermentation. Compared with the HT1 inoculation, the HT1 + EN inoculation did not enhance lactic fermentation after ensiling for 1 day but enhanced lactic fermentation after ensiling from 1 to 60 days. This indicated that, as long as HT1 was provided sufficient sugars that could be derived from cellulase-degrading lignocellulose, HT1 could restrain clostridial fermentation and improve the fermentation quality of high-moisture alfalfa silage when ensiling for prolonged periods of time. Compared with the EN-treated silage, the HT1 + EN-treated silage exhibited more vigorous lactic fermentation at the final ensiling time. This outcome did not verify that HT1 became the dominant species but it does suggest that HT1 had the ability to shift the inherent heterolactic fermentation into homolactic fermentation when the silage contained sufficient sugar. This was in contrast to the results of Lynch et al. [12], who found that inherent heterolactic fermentation in untreated alfalfa silage is difficult to change by adding cellulase combined with LAB after ensiling for 70 days. The inconsistent results from different studies were due to differences in the exogenous LAB inoculates. In the present study, *L. lactis* was a homofermentative LAB, while inoculates in the report of Lynch et al. contained heterofermentative LAB, *Lactobacillus buchneri* [12], which can degrade lactic acid to acetic acid, primarily after ensiling for 45 days [48].

HT2 considerably improved the fermentation quality of high-moisture alfalfa silage compared with the control silage in the present study. This was attributed to the fact that HT2 could secrete endoglucanase, cellobiohydrolase, and β-glucosidase to synergistically degrade lignocellulose into sufficient sugars utilizable by *L. lactis* to promote lactic fermentation. Similar results were found in the report of Li et al., who found that inoculation with a rumen *Enterococcus* strain resulted in the degradation of lignocellulose, which increased residual sugars, promoted lactic fermentation and preserved *Pennisetum sinese* silage as a biomass feedstock [4]. Compared with EN and HT1, the effect of promoting lactic fermentation and degrading lignocellulose into sugars was further enhanced after ensiling for 60 days. This outcome resulted from the dual function: HT2 secreted cellulase to degrade lignocellulose into sugars and promoted homolactic fermentation of itself. Moreover, HT2 degraded lignocellulose into more sugars utilizable by *L. lactis,* which not only enhanced fermentation of itself but also the high reserved sugars could benefit the subsequent biofuel production. A similar outcome was found in the report of Li et al., who found a high reserve of sugars in cellulolytic rumen *Enterococcus* strain-treated silage [4]. Kitamoto et al. reported that high preservation of soluble nonstructural carbohydrates deliver energy sources to microorganisms for maximally improving biofuel production, because they are apt to be converted into biofuel after the pretreatment process [6]. Moreover, HT2 had a lower consumption of sugars than HT1 + EN during 60 days of ensiling. According to the insignificant difference in the contents of lactic acid and acetic acid in HT2 and HT1 + EN after ensiling for 60 days, it was inferred that HT2 had a better ability to convert sugar to acid. Furthermore, HT2 more strongly degraded lignocellulose to produce xylose compared with the control and other treatments, which was verified by the decrease in hemicellulose and high residual xylose content during ensiling from 18 to 60 days. Xylose became one of the main sugars (45.2% in WSC) after prolonged ensiling, since xylose was hardly metabolized by the host *L. lactis* [49]. It can be inferred that the mechanism of hemicellulose degradation in the HT2-treated silage was enzymatic activity, acidolysis and microbial activity, which agreed with the outcome in the report of Dewar [50], who found that enzymolysis and acidolysis of hemicellulose after increasing nonstructural carbohydrates could occur effectively at a low pH. Therefore, HT2 achieved the intended functions of a highly efficient degrading lignocellulose and well-ensiling high-moisture alfalfa silage as a biomass feedstock.

## Conclusions

This is the first report in which engineered *L. lactis* strains with the *bgl*1, *cbh*2 and *egl*3 genes of *T. reesei* were successfully constructed and then extracellularly secreted endoglucanase, cellobiohydrolase and β-glucosidase. Ensiling experiments verified that the combination of transgenically engineered *L. lactis* strains ensiled high-moisture alfalfa silage efficiently, indicated by a lower pH and ammonia-N content, without butyric acid and a higher quality score compared with the untreated silage after ensiling for 60 days. The effects of transgenically engineered *L. lactis* strains on enhancing the conversion of lignocellulose to sugars were better than the combination of cellulase and wild-type *L. lactis* subsp. *lactis* MG1363, indicated by fewer structural carbohydrates and more available sugars in the silage treated with transgenically engineered *L. lactis* strains after ensiling for 60 days. Therefore, a new strategy for enhancing lignocellulosic degradation in high-moisture alfalfa was obtained by a bioprocess with transgenically engineered *L. lactis* strains, which could aid in the development of alfalfa as a biomass feedstock and promote second-generation biofuel development in the rainy season.

## Additional file

**Additional file 1.** Images of silages after silo opening. Control, silage treated without additives; EN, cellulase; HT1, wild-type *L. lactis* subsp. *lactis* MG1363; HT1 + EN, combination of HT1 and EN; HT2, combination of transgenically engineered *L. lactis* strains HT1*/*pMG36e-*usp*45-*bgl*1, HT1*/*pMG36e-*usp*45-*cbh*2, and HT1*/*pMG36e-*usp*45-*egl*3.

## Abbreviations

ADF: Acid detergent fibre
ADL: Acid detergent lignin
CMC: Carboxymethyl cellulose
Control: Silage treated without additives
DH5α: *Escherichia coli* DH5α
DM: Dry matter
EN: Cellulase
FC: Fermentation coefficient
FM: Fresh matter
HT1: Wild-type *L. lactis* subsp. lactis MG1363
HT1 + EN: Combination of HT1 and EN
HT2: Combinati on of transgenically engineered *L. lactis* strains HT1*/*pMG36e-*usp*45-*bgl*1, HT1*/*pMG36e-*usp*45-*cbh*2, and HT1*/*pMG36e-*usp*45-*egl*3
LA/AA: Ratio of lactic acid to acetic acid
LAB: Lactic acid bacteria
N: Nitrogen
NDF: Neutral detergent fibre
SDS-PAGE: Sodium dodecyl sulphate–polyacrylamide gel electrophoresis
SEM: Standard error of the means
*usp*45: Signal peptide of Usp45
WSC: Water-soluble carbohydrates
