# Supplementary material for: Enhancement of lignocellulosic degradation in high-moisture alfalfa via anaerobic bioprocess of engineered Lactococcus lactis with the function of secreting cellulase
Source: Biotechnol Biofuels. 2019 Apr 17;12:88. doi: 10.1186/s13068-019-1429-4 (PMC6469111; doi:10.1186/s13068-019-1429-4)
Supplement: Supplementary file 1 — Additional file 1. Images of silages after silo opening. Control, silage treated without additives; EN, cellulase; HT1, wild-type L. lactis subsp. lactis MG1363; HT1 + EN, combination of HT1 and EN; HT2, combination of transgenically engineered L. lactis strains HT1/pMG36e-usp45-bgl1, HT1/pMG36e-usp45-cbh2, and HT1/pMG36e-usp45-egl3. [file 13068_2019_1429_MOESM1_ESM.docx]

**Additional files**
